# Predicted long-term antibody persistence for a tick-borne encephalitis vaccine: results from a modeling study beyond 10 years after a booster dose following different primary vaccination schedules

**DOI:** 10.1080/21645515.2019.1700712

**Published:** 2020-01-17

**Authors:** Marco Costantini, Andrea Callegaro, Jiří Beran, Valérie Berlaimont, Ilaria Galgani

**Affiliations:** a GSK, Siena, Italy; b GSK, Rixensart, Belgium; c Vaccination and Travel Medicine Centre, Hradec Králové, Czechia; d Department for Tropical, Travel Medicine and Immunization, Institute for Postgraduate Medical Education in Prague, Prague 10, Czechia; e GSK, Wavre, Belgium

**Keywords:** Tick-borne encephalitis, polygeline-free inactivated TBE vaccine, antibody persistence, statistical model, power-law model

## Abstract

In tick-borne encephalitis (TBE)-endemic regions, long-term vaccination programs are efficient in preventing the disease. A booster dose of a polygeline-free inactivated TBE vaccine (*Encepur Adults*, GSK), administered approximately 3 years post-primary vaccination according to 1 of 3 licensed vaccination schedules in adults and adolescents, resulted in antibody persistence for 10 years post-boosting. We used different power-law models (PLMs) to predict long-term persistence of anti-TBE virus neutralization test (NT) antibody titers over a period of 20 years post-booster dose, based on individual antibody NT titers measured for 10 years post-booster vaccination. The PLMs were fitted on pooled data for all vaccine schedules. A mean NT titer of 261 (95% prediction interval: 22–3096), considerably above the accepted threshold of protection (NT titers ≥10), was predicted 20 years post-booster vaccination with TBE vaccine. Our modeled data suggest that the intervals of booster doses could be increased without compromising protection against TBE.

## Introduction

Tick-borne encephalitis (TBE), an infectious disease of the central nervous system, is caused by the TBE virus (TBEV) of the *Flavivirus* genus, transmitted mainly through *Ixodes* ticks. In the European Union, TBE is notifiable since 2012; notification rates were the same in 2014 and 2015 (0.4/100,000 population),^1^ although emerging data from 2016 illustrate an increase in the number of cases for eight countries.^2^

The non-specificity of clinical symptoms in the initial viremic phase and the lack of effective treatment make TBE very difficult to manage. This can lead to long-term sequelae in more than 30% of cases.^3^ Case-fatality rates differ according to the TBEV subtype, but can reach up to 40% for the Far-Eastern subtype, while being significantly lower for the Siberian (6–8%), and European (≤1.4%) variation.^3,4^ Several vaccines, based on different strains of TBEV, are available and considered effective in preventing TBE.^4^

The polygeline-free inactivated TBE vaccine (*Encepur Adults*, GSK), targeting the European subtype, is licensed in Europe for use in adults and adolescents ≥12 years of age and has been shown to be immunogenic and well tolerated,^5,6^ with persistently high antibody titers induced up to 10 years following a first booster vaccination.^7,8^ The TBE vaccine has also been reported to induce antibodies against various Asian TBEV isolates.^4,9^

Although there is no established correlate of protection against TBE, the World Health Organization supports the use of a surrogate marker of protection represented by the presence of circulating antibodies against TBEV at or above a locally agreed concentration considered as clinically meaningful.^4,5^ In clinical studies conducted with the currently licensed polygeline-free formulation, antibody response elicited by the TBE vaccine was measured by a validated in-house neutralization test (NT), for which anti-TBEV NT titers ≥2 and ≥10 were chosen as thresholds indicative of seropositivity and of a clinically meaningful antibody response, respectively.^10,11^

The World Health Organization recommends vaccination against TBE for individuals of all ages living in highly endemic areas and to targeted cohorts (>50–60-year-olds) in low-to-moderate-endemic regions, as well as to travelers planning outdoor activities in TBE-endemic areas.^4^ Several TBE-endemic European countries recommend routine vaccination as a three-dose primary series according to one out of three different approved schedules, followed by repeated booster doses.^12^ However, the optimal intervals for booster vaccination necessary to ensure long-term protection against TBE are still being debated, as only a few studies reported on long-term immunogenicity of available vaccines.^8,13^ A recent study demonstrated that ≥90% of individuals vaccinated with the TBE vaccine maintained NT titers ≥10 up to 10 years after a first booster dose, regardless of age and primary vaccination schedule received.^8^ Moreover, no TBE infections occurred during this period.^8^

With the aim of estimating the evolution of the immune response over time beyond available clinical data, we modeled anti-TBEV NT antibody levels up to 20 years post-first boosting following primary vaccination with one of the 3 licensed schedules.^11^ We used several power-law models (PLMs), based on antibody levels measured in two extension studies in participants followed 5^7^ and 10 years^8^ post-booster dose to predict the development of antibody levels beyond the time period covered by clinical trials.

**Figure 1.**
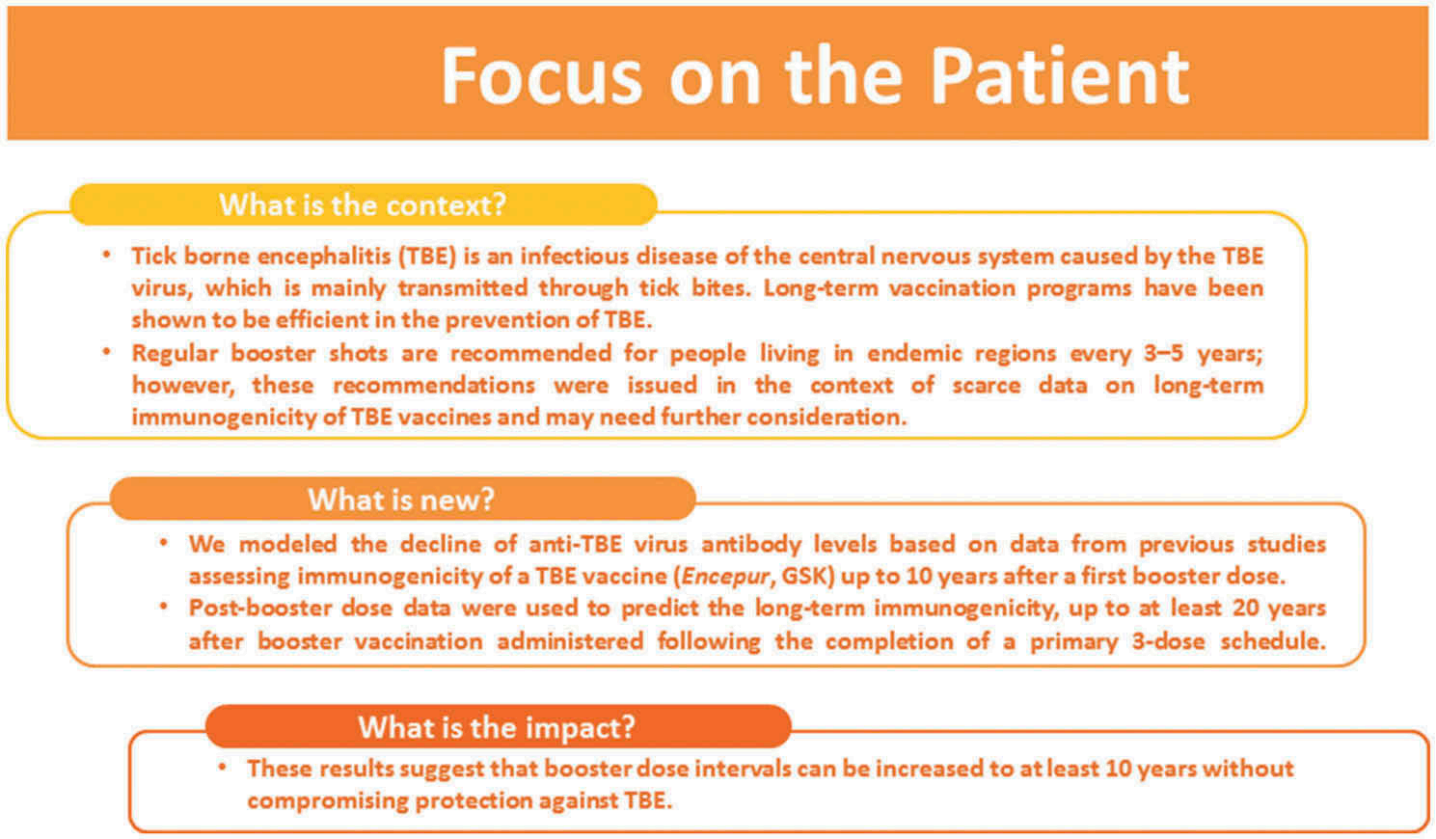
Focus on the patient section.

A summary contextualizing the results, the potential clinical research relevance and the impact of our study is displayed in Figure 1, for the benefit of health-care practitioners.

## Methods

In the primary study, a randomized trial conducted in Czechia, adolescents and adults ≥12 years old received three primary doses of the TBE vaccine (*Encepur*) according to a licensed (rapid, conventional or accelerated conventional) or an unlicensed (modified conventional) vaccination schedule.^11^ In the first extension study (NCT00387634), 283 participants received a booster dose at study entry, 3 years post-primary vaccination. Additionally, 40 participants who had received a booster dose 12–18 months post-primary were enrolled; all were followed up for 5 years.^7^ Of these, 201 individuals who had received one of the licensed vaccination schedules were followed up in the second extension study (NCT01562444) from year 6 to 10 post-booster dose.^8^

The current analysis was conducted using individual anti-TBEV NT titers of adolescents and adults vaccinated with one of the three prime-boost vaccination schedules, measured once a year, for up to 10 years following the booster dose. All available data from participants in each of the primary and extension studies were used. The assay used for the evaluation of NT titers was previously described.^14^ The use of the data from the previously collected samples was covered by approval from the Ethics Committee of the University Hospital Hradec Králové.

We used several different PLMs to model the data. The PLM has previously been employed to predict long-term antibody persistence post-vaccination against the human papillomavirus.^15,16^ Briefly, the model accounts for an exponential rate of B-cell decay to estimate the persistence of antibody levels over time, following vaccination.

A more sophisticated adaptation of the PLM is the piecewise PLM (PPLM). The PPLM combines multiple PL functions, each fitted to different, consecutive intervals of data, which allows for more flexible estimation of the evolution of antibody levels over the entire period of time. The monotone PPLM (MPPLM) further restrained these functions from increasing in value to exclude a potentially biologically not meaningful increase in antibody level over time. Finally, we considered the extended PLM (EPLM) proposed by Fraser et al. This model is an extension of the PLM which accounts for two populations of B-cells involved in a long-term antibody plateau: activated and memory B-cells.^16^

The models were compared using the classic model selection based on the Akaike information criterion (AIC). Moreover, models were compared based on prediction error. To that end, a separate calculation was done in which the two last years of measured data were not considered. A sensitivity analysis excluding outliers was also performed, with outliers identified by the standard interquartile range rule (1.5 × IQR).

The values predicted by the models were then compared with the measured values. All predicted values were reported as geometric means.

All statistical analyses were performed using the PROC NLMIXED subroutine in Statistical Analysis Systems (SAS). Formulas, SAS syntax, and estimated parameters for each model are provided in the Supplementary Material.

## Results

**Figure 2.**
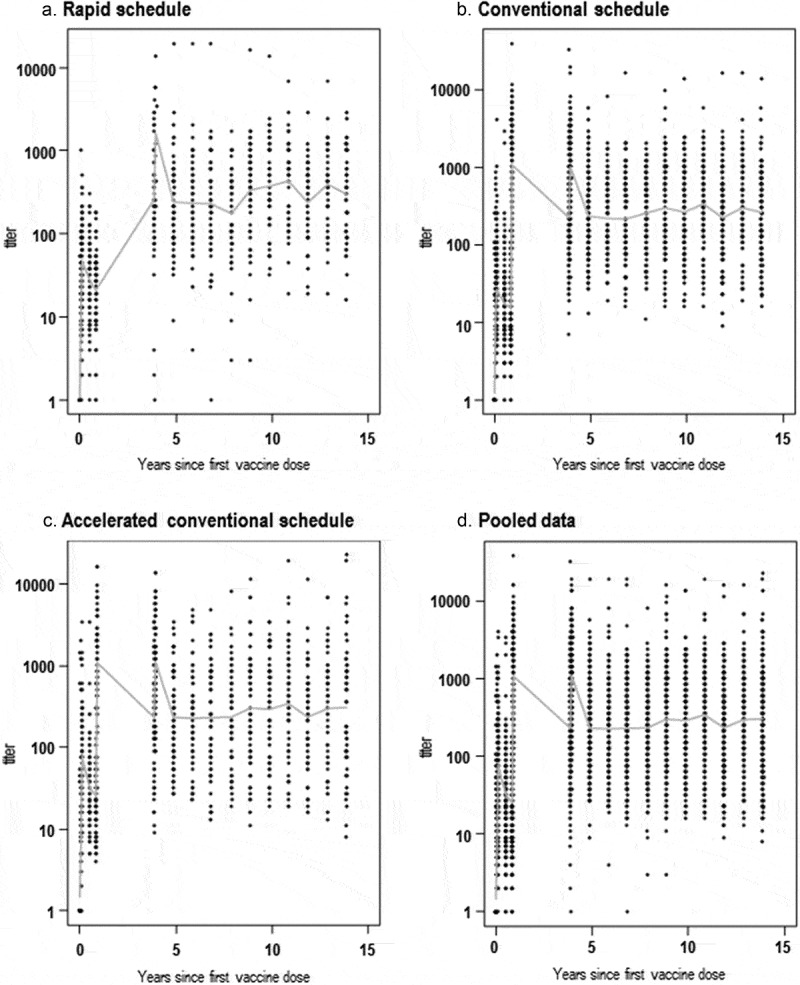
Distribution of measured anti-TBEV antibody NT titers over 15 years post-first dose of the TBE vaccine, administered according to the rapid (a), conventional (b), accelerated conventional (c) primary schedules and pooled data (d).

For all three primary vaccination schedules (rapid, conventional and accelerated conventional), a similar yearly distribution of anti-TBEV antibody mean NT titers was observed following the booster dose (Figure 2). Only 14 outliers were identified throughout the 10-year follow-up (Supplementary Figure 1).

* Data for year 4 since the first vaccine dose correspond to 21 days post-booster dose, administered 3 years post-primary vaccination (conventional and accelerated conventional schedule) or 1.5–2 years post-booster dose, administered at 12–18 months post-primary vaccination (rapid schedule). Nine of the 49 individuals receiving the rapid schedule were administered the booster dose at 3 years post-primary vaccination, causing the artificial peak in NT titers at year 4, but these data were compensated for in the modeling.

**Figure 3.**
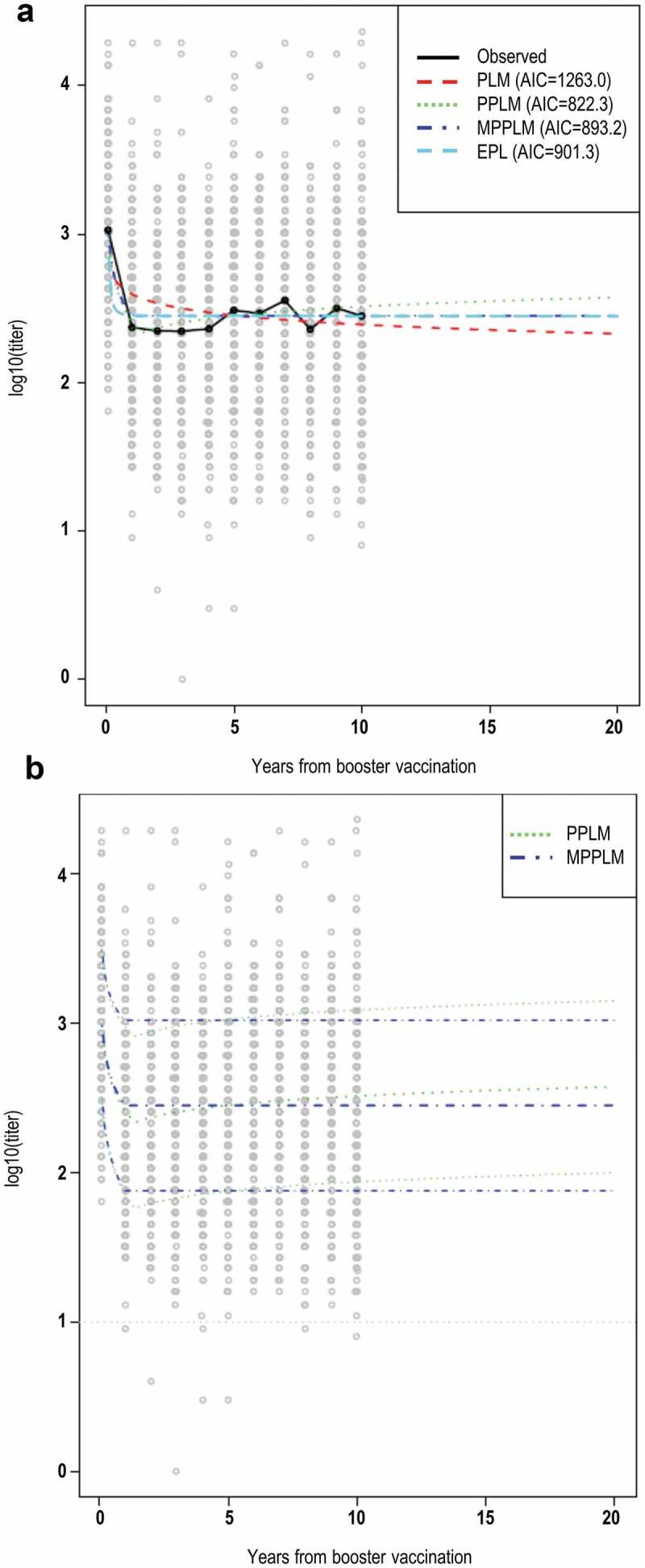
Mean anti-TBEV antibody NT levels over 20 years post-booster dose, as predicted by the different models (a) and prediction intervals for PPLM and MPPL (b).

Note: Curves indicate the observed and predicted mean NT titer (panel A) and predicted mean NT titer with upper and lower limits of the 95% prediction interval (panel B). The dotted gray line (panel B) indicates the threshold considered as indicative of protection against TBE (NT titer of 10).

When the PLM was adjusted to account for the vaccination schedule using post-booster data only, the effect of the primary schedule was found to be statistically non-significant (*p*-value = 0.11). Therefore, all models were applied on pooled data from participants receiving any of the three vaccination schedules and considering the timing of the booster dose (i.e., year 3 post-first primary dose for the majority of participants) as the starting time point for the prediction (Figure 3).

All models showed an initial decline until year 1 post-booster dose, with values stabilizing after this timepoint. The PLM-predicted a mean anti-TBEV antibody NT titer decline from 386 at year 1 post-booster dose to 197 at year 20 post-booster dose (Figure 3). The PPLM showed a slight increase after the initial drop, predicting a titer of 349 at year 20 post boosting. The MPPL predicted the most stable antibody levels, with a titer of 261 at year 20 post booster; the same value was predicted by the EPLM. The modeled prediction intervals show that >95% of patients are predicted to have antibody NT titer levels >10 within a timeframe of 20 years post-booster dose (Table 1).

**Table 1.** Mean anti-TBEV antibody NT titers at 20 years post-booster vaccination as predicted by power law models.

Model	Mean NT titer	95% prediction interval
PLM	197.60	13.17–2964.23
PPL	349.83	29.31–4175.42
MPPL	261.49	22.08–3096.31
EPL	261.17	22.05 3092.98

TBEV, tick-borne encephalitis virus; NT, neutralization test; PLM, power law model; PPLM, piecewise PLM; MPPLM, monotone PPLM; EPLM, extended PLM.

According to the AIC the model providing the best fit is the PPLM. This was supported by the evaluation of the prediction error.

The sensitivity analysis showed that the outliers had no impact on the results of the pooled data modeling (data not shown).

## Discussion

To our knowledge, this is the first study modeling long-term antibody persistence after vaccination against TBE, providing modeled data beyond the 10 years post-first booster period investigated to date. Twenty years post-booster vaccination with the TBE vaccine, different models predict a mean NT titer considerably above the established threshold of 10 for more than 95% of individuals who were included in these analyses. Therefore, sufficient protection against TBE is predicted for at least 20 years after booster vaccination with the TBE vaccine, when any of the licensed primary three-dose schedules is completed and a booster dose is administered at 1 or 3 years post-primary vaccination, depending on the primary schedule.

When considering the follow-up of adolescents and adults who received the full primary vaccination series and a booster dose, no consistent decline in antibody NT titers was measured over a period of 10 years. For instance, at 5 years post-booster dose, the observed geometric mean titers ranged between 300 and 429 across groups,^7^ while at 10 years post-booster, they were 166–245 in the total-vaccinated cohort and 260–307 in the per-protocol set.^8^ Of note, over 10 years of follow-up post-boosting, no consistent decline in geometric mean NT titers was observed within the same age groups, although smaller overall values were observed for participants receiving the booster dose at 50–59 and ≥60 years of age than at 15–49 years of age.^8^ However, the comparison was hindered by a relatively small sample size for the ≥60-year age group, which also applied for our analysis. Therefore, analyses were not performed by age stratum.

According to the AIC, the best model for the provided data was the PPLM, which predicted an increase of antibody levels over the timeframe considered. This increase simulates the slight increase in antibodies observed in the measured data obtained during the trials. One possibility is that exposure to or vaccination against other flaviviruses (such as yellow fever, dengue or Japanese encephalitis) could result in antibody formation with a certain cross-reactivity within the assay; although this is unlikely for NT, which was shown to distinguish between pathogens belonging to the TBEV family.^17^ An exposure to TBEV itself appears unlikely, since in the case of infection a more sustained increase in antibody levels would be expected. However, the predicted values as well as the prediction interval indicate that participants not showing elevated results due to potential cross-reactivity or high baseline levels would still have NT titers consistently above threshold levels after 20 years post-boosting.

Our modeled data suggest that the interval between booster doses currently recommended for immunization against TBE (every 3–5 years)^12^ may be increased without compromising the expected protection, thus potentially lowering associated costs from both the societal and patient perspective. However, this remains to be confirmed by further empirical evidence and results from future studies following up vaccinated individuals will provide data against which the predicted rate of antibody titer decline could potentially be validated. Evaluation up to 15 years post-booster dose is currently ongoing as part of a third extension study (NCT03294135).

While generally, results of a statistical analysis may vary depending on the model used, a strength of this analysis is the use of several different models leading to similar results demonstrating the robustness of the prediction. However, the analysis has also several potential limitations. Among those, one of the most important is the assumption about the dynamics and decline of B- and T-cells, as the statistical analysis cannot be the sole driver for the conclusions. Another potential limitation is the fact that a drop in NT titers was not predicted by any of the models, as the observed data used as basis for this modeling exercise showed a steady trend beyond one year post-booster dose, without reaching the turning point where antibodies started to fall, which made it impossible to estimate the antibody half-life. Due to the relatively small sample size for the older age range, in particular, the ≥60-year age group, the analyses were not carried out by age strata; potential extrapolation of results from the pooled age groups to individuals ≥60 years of age should therefore be interpreted with caution. Moreover, these results are specific to the TBE vaccine used, and cannot be generalized to other vaccines.

In conclusion, the predicted antibody persistence was considerably above the surrogate marker of protection (NT titers ≥10) up to 20 years post-booster vaccination with the TBE vaccine. Our results suggest that booster schedules could be increased in the future, although our modeled estimates still need to be confirmed by field data.

## Supplementary Material

Supplemental Material

## Abbreviations

AIC: Akaike information criterion
CI: Confidence interval
IQR: Interquartile range
NT: Neutralization test
PLM: Power-law model
TBE: Tick-borne encephalitis
TBEV: Tick-borne encephalitis virus;
TBE vaccine: Polygeline-free inactivated TBE vaccine

## Data sharing statement

Anonymized individual participant data from the described studies plus the annotated case report form, protocol, reporting and analysis plan, data set specifications, raw dataset, analysis-ready dataset, and clinical study report are available for research proposals approved by an independent review committee. Proposals should be submitted to www.clinicalstudydatarequest.com. A data access agreement will be required.

## Trademark statement

*Encepur Adults* is a trademark owned by the GSK group of companies.

## References

[1] European Centre for Disease Prevention and Control. Tick-borne encephalitis. ECDC. Annual Epidemiological Report for 2015. Stockholm: ECDC; 2018 [accessed 2019 819]. http://ecdc.europa.eu/sites/portal/files/documents/AER_for_2015-tick-borne-encephalitis.pdf.

[2] Kunze U; The ISW-TBE. Report of the 19th annual meeting of the International Scientific Working Group on Tick-Borne Encephalitis (ISW-TBE) - TBE in a changing world. Ticks Tick Borne Dis. 2018;9(2):146–50. doi:10.1016/j.ttbdis.2017.08.009. 28918352

[3] Lindquist L. Tick-borne encephalitis. Handb Clin Neurol. 2014;123:531–59. doi:10.1016/b978-0-444-53488-0.00025-0. 25015503

[4] Vaccines against tick-borne encephalitis: WHO position paper. Wkly Epidemiol Rec. 2011;86(24):241–56. 21661276

[5] Kollaritsch H, Krasilnikov V, Holzmann H, Karganova G, Barrett A, Süss J.; World Health Organization (WHO). Background document on vaccines and vaccination against tick–borne encephalitis. Geneva (Switzerland): WHO Strategic Advisory Group of Experts on Immunization; 2011 [accessed 2019 819]. http://www.who.int/immunization/sage/6_TBE_backgr_18_Mar_net_apr_2011.pdf.

[6] Galgani I, Bunge EM, Hendriks L, Schludermann C, Marano C, De Moerlooze L. Systematic literature review comparing rapid 3-dose administration of the GSK tick-borne encephalitis vaccine with other primary immunization schedules. Expert Rev Vaccines. 2017;16(9):919–32. doi:10.1080/14760584.2017.1358620. 28770638

[7] Beran J, Douda P, Gniel D, Zent O. Long-term immunity after vaccination against tick-borne encephalitis with Encepur using the rapid vaccination schedule. Int J Med Microbiol. 2004;293(Suppl 37):130–33. doi:10.1016/S1433-1128(04)80023-8. 15146994

[8] Beran J, Lattanzi M, Xie F, Moraschini L, Galgani I. Second five-year follow-up after a booster vaccination against tick-borne encephalitis following different primary vaccination schedules demonstrates at least 10 years antibody persistence. Vaccine. 2019;37(32):4623–29. doi:10.1016/j.vaccine.2017.12.081. 29397225

[9] Leonova GN, Pavlenko EV. Characterization of neutralizing antibodies to far eastern of tick-borne encephalitis virus subtype and the antibody avidity for four tick-borne encephalitis vaccines in human. Vaccine. 2009;27(21):2899–904. doi:10.1016/j.vaccine.2009.02.069. 19366574

[10] Paulke-Korinek M, Rendi-Wagner P, Kundi M, Laaber B, Wiedermann U, Kollaritsch H. Booster vaccinations against tick-borne encephalitis: 6 years follow-up indicates long-term protection. Vaccine. 2009;27(50):7027–30. doi:10.1016/j.vaccine.2009.09.068. 19786143

[11] Schöndorf I, Beran J, Cizkova D, Lesna V, Banzhoff A, Zent O. Tick-borne encephalitis (TBE) vaccination: applying the most suitable vaccination schedule. Vaccine. 2007;25(8):1470–75. doi:10.1016/j.vaccine.2006.10.028. 17196713

[12] European Centre for Disease Prevention and Control. Vaccine scheduler. Tick-Borne Encephalitis: Recommended Vaccinations; [accessed 2019 819]. http://vaccine-schedule.ecdc.europa.eu/Scheduler/ByDisease?SelectedDiseaseId=27&SelectedCountryIdByDisease=−1.

[13] Konior R, Brzostek J, Pöllabauer EM, Jiang Q, Harper L, Erber W. Seropersistence of TBE virus antibodies 10 years after first booster vaccination and response to a second booster vaccination with FSME-IMMUN 0.5mL in adults. Vaccine. 2017. doi:10.1016/j.vaccine.2017.03.059. 28545923

[14] Klockmann U, Bock HL, Kwasny H, Praus M, Cihlová V, Tomková E, Křivanec K. Humoral immunity against tick-borne encephalitis virus following manifest disease and active immunization. Vaccine. 1991;9(1):42–46. doi:10.1016/0264-410X(91)90315-W. 2008800

[15] David MP, Van Herck K, Hardt K, Tibaldi F, Dubin G, Descamps D, Van Damme P. Long-term persistence of anti-HPV-16 and -18 antibodies induced by vaccination with the AS04-adjuvanted cervical cancer vaccine: modeling of sustained antibody responses. Gynecol Oncol. 2009;115(3 Suppl):S1–6. doi:10.1016/j.ygyno.2009.01.011. 19217149

[16] Fraser C, Tomassini JE, Xi L, Golm G, Watson M, Giuliano AR, Barr E, Ault KA. Modeling the long-term antibody response of a human papillomavirus (HPV) virus-like particle (VLP) type 16 prophylactic vaccine. Vaccine. 2007;25(21):4324–33. doi:10.1016/j.vaccine.2007.02.069. 17445955

[17] Holzmann H, Kundi M, Stiasny K, Clement J, McKenna P, Kunz C, Heinz FX. Correlation between ELISA, hemagglutination inhibition, and neutralization tests after vaccination against tick-borne encephalitis. J Med Virol. 1996;48(1):102–07. doi:10.1002/(sici)1096-9071(199601)48:1<102::aid-jmv16>3.0.co;2-i. 8825718

