# Supplementary material for: Predicted long-term antibody persistence for a tick-borne encephalitis vaccine: results from a modeling study beyond 10 years after a booster dose following different primary vaccination schedules
Source: Hum Vaccin Immunother. 2020 Jan 17;16(9):2274–9. doi: 10.1080/21645515.2019.1700712 (PMC7553683; doi:10.1080/21645515.2019.1700712)
Supplement: Supplemental Material [file KHVI_A_1700712_SM0936.zip › Suppl text_Costantini et al_HVI 2019-revised.docx]

# Supplementary Material. Statistical methods

Model definitions

Notation: *Ab_ij,_* antibody titer for individual *i* (*i* = 1, ….., n) collected at visit *j*, (*j* = 1, ….., n_i_) at time *t_ij_* from the visit before booster dose administration.

Power law model:

*log*_10_*(Ab_ij_)=b_0i_+b_1i_log*_10_*(t_ij_)+ε_ij_,*

where *b_0i_* and *b_1i_* are correlated random intercept and slope.

Piecewise power law model:

$${log}_{10}\left( Ab_{ij} \right)=\left\{ \begin{aligned} b_{0i}+b_{1i}{log}_{10}\left( t_{ij} \right)+\varepsilon_{ij}, &if t_{ij}\leq\delta\\ b_{0i}+b_{1i}{log}_{10}\left( t_{ij} \right)+b_{2i}\left( {log}_{10}\left( t_{ij} \right)-\delta\right)+ \varepsilon_{ij}, if t_{ij}>\delta\end{aligned} \right.$$

where *b_0i_* and *b_1i_* are correlated random effects and 𝑏_2𝑖_ is a fixed effect (𝑏_2_).

Monotone piecewise power law model:

a piecewise power law model with the following constrains: $E\left( b_{1i} \right)\leq0; E\left( b_{2i} \right)\leq0$.

Extended power law model:

$${log}_{10}\left( Ab_{ij} \right)=k_{i}+{log}_{10}\left( \left( 1-\pi\right){t_{ij}}^{-a_{i}}+\pi\right)+\varepsilon_{ij}$$

where π is the relative level of antibody produced in the long-term memory plateau.

**SAS syntax**

Power-law model

**PROC** **NLMIXED** DATA=dd cov;

PARMS se=**0.2358** a0 = **0.3689** k0 = **3.2581** sk= -**0.4182** sa=-**0.1170** ska=**0.005832** ;

q = (**1**/t)**a;

lg=log10(q);

mean = k + lg;

MODEL log_val ~ NORMAL(mean,se*se);

RANDOM k a ~ NORMAL([k0,a0], [sk*sk, ska, sa*sa]) SUBJECT=pid out = indiv;

predict mean out=pred_pl;

**RUN**;

Piecewise power law model

**proc** **nlmixed** data=dd;

parms se=**0.3** a0 = **2** b0 = -**1** b2=**0** delta=**0** sk=**0.5** sa=**0.5** ska=**0.01**;

Xpart = a1 + b1*logtime;

IF (logtime > delta) THEN DO;

Xpart = a1 + delta*(b1-b2) + b2*logtime;

end;

model log_val~normal(Xpart,se);

RANDOM a1 b1 ~ NORMAL([a0,b0], [sk*sk, ska, sa*sa]) SUBJECT=pid;

predict Xpart out=pred_ppl;

**run**;

Monotone piecewise power law model

**proc** **nlmixed** data=dd;

parms se=**0.3** a0 = **2** b0 = -**1** b2=-**1** delta=**0** sk=**0.5** sa=**0.5** ska=**0.01**;

bounds b2<=**0**;

Xpart = a1 + b1*logtime;

IF (logtime > delta) THEN DO;

Xpart = a1 + delta*(b1-b2) + b2*logtime;

end;

model log_val~normal(Xpart,se);

RANDOM a1 b1 ~ NORMAL([a0,b0], [sk*sk, ska, sa*sa]) SUBJECT=pid out = indiv;

predict Xpart out=pred_mppl;

**run**;

Extended power law model

**PROC** **NLMIXED** DATA=dd;

PARMS se=**0.2854** a0 = **0.2** k0 = **2.8** sk= **0.4** sa=**0.4** pi=**0.8** ska=**0**;

*bounds 0 <= pi <=1;

mean = k + log10((**1**-pi)*(**1**/t)**a+pi);

MODEL log_val ~ NORMAL(mean,se*se);

RANDOM k a ~ NORMAL([k0,a0], [sk*sk, ska, sa*sa]) SUBJECT=pid out = indiv;

predict mean out=pred_epl;

**RUN**;

**Estimated parameters**

Power-law model

| Parameter | Estimate | SE | DF | t Value | Pr > \|t\| | 95% confidence interval | |
| --- | --- | --- | --- | --- | --- | --- | --- |
| se | 0.2629 | 0.00448 | 210 | 58.64 | <.0001 | 0.2541 | 0.2718 |
| a0 | 0.2069 | 0.01373 | 210 | 15.07 | <.0001 | 0.1798 | 0.2339 |
| k0 | 2.5649 | 0.03565 | 210 | 71.95 | <.0001 | 2.4946 | 2.6352 |
| sk | -0.5057 | 0.02581 | 210 | -19.59 | <.0001 | -0.5565 | -0.4548 |
| sa | 0.1243 | 0.01636 | 210 | 7.60 | <.0001 | 0.09206 | 0.1566 |
| ska | -0.0278 | 0.00722 | 210 | -3.85 | 0.0002 | -0.04204 | -0.01356 |

Piecewise power law model

| Parameter | Estimate | SE | DF | t Value | Pr > \|t\| | 95% confidence interval | |
| --- | --- | --- | --- | --- | --- | --- | --- |
| se | 0.0568 | 0.00189 | 210 | 29.91 | <.0001 | 0.0530 | 0.0605 |
| a0 | 2.3827 | 0.04000 | 210 | 59.57 | <.0001 | 2.3039 | 2.4616 |
| b0 | -0.5570 | 0.2359 | 210 | -23.61 | <.0001 | -0.6035 | -0.5105 |
| b2 | 0.2073 | 0.02519 | 210 | 8.23 | <.0001 | 0.1576 | 0.2569 |
| delta | 0.1420 | 0.03116 | 210 | 4.56 | <.0001 | 0.0806 | 0.2035 |
| sk | 0.5316 | 0.0265 | 210 | 20.08 | <.0001 | 0.4794 | 0.5837 |
| sa | 0.1330 | 0.02335 | 210 | 5.70 | <.0001 | 0.0869 | 0.1790 |
| ska | 0.0487 | 0.0101 | 210 | 4.82 | <.0001 | 0.0288 | 0.0685 |

Monotone piecewise power law model

| Parameter | Estimate | SE | DF | t Value | Pr > \|t\| | 95% confidence interval | |
| --- | --- | --- | --- | --- | --- | --- | --- |
| se | 0.0594 | 0.0019 | 210 | 29.93 | <.0001 | 0.0555 | 0.0633 |
| a0 | 2.4128 | 0.08142 | 210 | 29.63 | <.0001 | 2.2523 | 2.5733 |
| b0 | -0.5322 | 0.0660 | 210 | -8.06 | <.0001 | -0.6623 | -0.4020 |
| delta | -0.0087 | 0.1353 | 210 | -0.06 | 0.9487 | -0.2754 | 0.2580 |
| ska | 0.0544 | 0.0139 | 210 | 3.90 | 0.0001 | 0.0269 | 0.0818 |
| sk | 0.5441 | 0.0301 | 210 | 18.06 | <.0001 | 0.4847 | 0.6035 |
| sa | 0.1260 | 0.03250 | 210 | 3.88 | 0.0001 | 0.06192 | 0.1901 |
| ska | 0.0543 | 0.0139 | 210 | 3.90 | 0.0001 | 0.0269 | 0.0818 |

Extended power law model

| Parameter | Estimate | SE | DF | t Value | Pr > \|t\| | 95% confidence interval | |
| --- | --- | --- | --- | --- | --- | --- | --- |
| se | 0.2434 | 0.0041 | 210 | 59.79 | <.0001 | 0.2354 | 0.2514 |
| a0 | 2.0026 | 0.2397 | 210 | 8.36 | <.0001 | 1.5301 | 2.4751 |
| k0 | 2.4231 | 0.0379 | 210 | 64.02 | <.0001 | 2.3485 | 2.4978 |
| sk | 0.5432 | 0.0270 | 210 | 20.12 | <.0001 | 0.4900 | 0.5964 |
| sa | 0.1968 | 0.0394 | 210 | 4.99 | <.0001 | 0.1191 | 0.2746 |
| pi | 0.9858 | 0.0090 | 210 | 109.92 | <.0001 | 0.9681 | 1.0034 |
| ska | -0.0758 | 0.0165 | 210 | -4.61 | <.0001 | -0.1082 | -0.0433 |
