# Supplementary figures and images for: Predicted long-term antibody persistence for a tick-borne encephalitis vaccine: results from a modeling study beyond 10 years after a booster dose following different primary vaccination schedules
Source: Hum Vaccin Immunother. 2020 Jan 17;16(9):2274–9. doi: 10.1080/21645515.2019.1700712 (PMC7553683; doi:10.1080/21645515.2019.1700712)

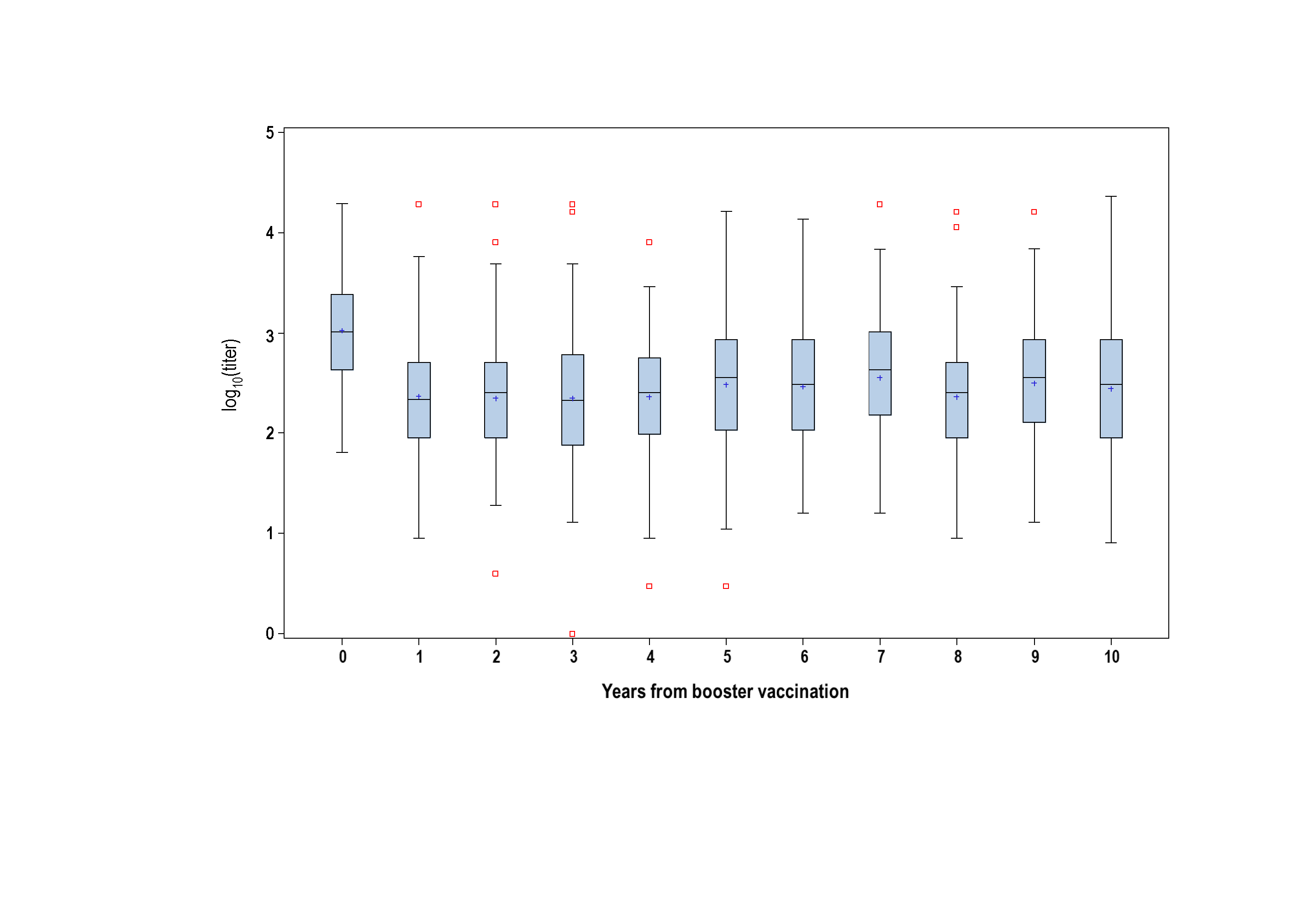

Supplement: Supplemental Material [file KHVI_A_1700712_SM0936.zip › Suppl Fig 1_Costantini et al_HVI2019.tiff]
